# Improved Tissue Processing in Esophageal Adenocarcinoma After Ivor Lewis Esophagectomy Allows Histological Analysis of All Surgically Removed Lymph Nodes with Significant Effects on Nodal UICC Stages

**DOI:** 10.1245/s10434-020-09450-1

**Published:** 2020-12-10

**Authors:** A. Quaas, H. Schloesser, H. Fuchs, T. Zander, C. Arolt, A. H. Scheel, J. Rueschoff, C. Bruns, R. Buettner, W. Schroeder

**Affiliations:** 1 Institute of Pathology, Gastrointestinal Cancer Group Cologne (GCGC), University Hospital Cologne, Cologne, Germany; 2 Department of General, Visceral, Cancer and Transplantation Surgery, University Hospital Cologne, Cologne, Germany; 3 Department of Internal Medicine I, University Hospital Cologne, Cologne, Germany; 4 Institute of Pathology, Nordhessen and Targos Molecular Pathology GmbH, Kassel, Germany

**Keywords:** Lymph nodes, Acetone compression, Esophageal adenocarcinoma, Esophagectomy, UICC stage

## Abstract

**Background:**

In esophageal carcinoma, the numbers of metastatic and total removed lymph nodes (LN) are well-established variables of long-term prognosis. The overall rate of retrieved LN depends on neoadjuvant treatment, the extent of surgical lymphadenectomy, and the modality of the pathological workup. The question in this study is whether technically extended histopathological preparation can increase the number of detected (metastatic) LN with an impact on nodal UICC staging.

**Patients and Methods:**

A cohort of 77 patients with esophageal adenocarcinoma was treated with Ivor Lewis esophagectomy including standardized two-field lymphadenectomy. The specimens were grossed, and all manually detectable LN were retrieved. The remaining tissue was completely embedded by the advanced “acetone compression” retrieval technique. The primary outcome parameter was the total number of detected lymph nodes before and after acetone workup.

**Results:**

A mean number of 23,1 LN was diagnosed after standard manual LN preparation. With complete embedding of the fatty tissue using acetone compression, the number increased to 40.5 lymph nodes (*p* < 0.0001). The mean number of metastatic LN increased from 3.2 to 4.2 nodal metastases following acetone compression (*p* < 0.0001). Additional LN metastases which caused a change in the primary (y)pN stage were found in ten patients (13.0%).

**Conclusions:**

Advanced lymph node retrieval by acetone compression allows a reliable statement on the real number of removed LN. Results demonstrate an impact on the nodal UICC stage. A future multicenter study will examine the prognostic impact of improved lymph node retrieval on long-term oncologic outcome.

**Supplementary Information:**

The online version of this article contains supplementary material available at 10.1245/s10434-020-09450-1.

Multimodal treatment including transthoracic esophagectomy is generally accepted as standard care for patients with locally advanced but curable esophageal carcinoma.^1^ However, despite improvements in surgical techniques and perioperative management, as well as advancement of radiation and chemotherapy protocols, overall prognosis remains limited with 5-year survival rates ranging from 5 to 25%. In this context, oncologic outcome predominantly depends on the final histopathological TNM staging, response to neoadjuvant treatment and complete resection of the primary tumor (R status).^2^

For nodal status, the number of malignant LN is still the most important oncological variable, and histopathological pN, as well as corresponding UICC staging, are primarily based on this parameter.^3^ In addition, extracapsular involvement of metastatic nodes also demonstrated a negative prognostic value.^4^ Besides the number of metastatic LN, the total number of resected LN also has significant impact on long-term survival.^5^ These findings are the rationale for systematic lymphadenectomy as an inherent component of surgical procedure for esophageal cancer.

The number of histopathologically detected nodes is influenced by three distinct factors. First, due to the extensive submucosal lymphatic drainage, adeno- and squamous cell carcinoma exhibit a bidirectional lymphatic spread to mediastinal and abdominal nodes. To achieve complete clearance of possible nodal metastasis, a transthoracic surgical approach with extended lymphadenectomy of the mediastinum and upper abdomen (two-field LAD) is generally recommended and practiced by the majority of esophageal centers.^6^ Secondly, neoadjuvant radiochemotherapy and also chemotherapy lower the number of lymph nodes retrieved after surgical resection.^7^ Third, the postoperative pathological workup of the resected specimen itself influences the yield of total and metastatic nodes. Morphological studies on lymph node size demonstrate that the vast majority of resected nodes are < 4 mm with no correlation between size and malignant involvement.^8^ Standard workup of an esophagectomy specimen comprises careful preparation of removed fatty and soft tissue attached to the esophageal tube and upper stomach to visualize macroscopically recognizable or palpable lymph nodes, followed by hematoxylin–eosin staining and counting the number of actual lymph nodes present by light microscopy.

Due to the importance of the histopathological workup for node detection, this prospective observational study describes an innovative pathological method aiming to increase the lymph node yield from a standard esophagectomy specimen and analyzes the possible impact on pN and UICC staging of esophageal cancer.^9^

## Patients and Methods

### Study Population

This prospective observational study included all consecutive patients in the period from 03/2019 to 10/2019 who underwent esophagectomy for esophageal cancer at the Department of General, Visceral, Cancer, and Transplantation Surgery, University of Cologne. Of this study population, only patients with adenocarcinoma (AC) of the esophagus (Siewert AEG type I and II) who underwent standardized Ivor Lewis esophagectomy were included. In total, the cohort comprised *n* = 77 cases. The study was based on data obtained from a prospective database.

### Multimodal Treatment

All included patients underwent treatment with curative intent according to national guidelines.^10^ Patients with locally advanced carcinoma (cT3/4) received multimodal therapy, either as neoadjuvant chemoradiotherapy according to the CROSS protocol (5-week cycle of chemoradiation: 41.4 Gy, paclitaxel/carboplatin) or neoadjuvant chemotherapy based on the FLOT protocol (four 2-week cycles of docetaxel, oxaliplatin, fluorouracil, and leucovorin as a 24-h infusion, all on day 1).^11^^–^13

Patients with a primary tumor classified as cT1/2 or patients with multiple comorbidities were scheduled for primary esophagectomy.

All included patients underwent transthoracic en bloc esophagectomy with lymphadenectomy of the abdominal and mediastinal compartment (two-field lymphadenectomy, LAD). LN dissection of the abdominal compartment comprised groups no. 1, 2, and 3 (D1), as well as no. 7, 8, 9, and 11 (D2), according to the Japanese Society for Gastric Cancer.^14^ In the mediastinal compartment, LN of the lower mediastinum (nos. 108, 110, 112), tracheal bifurcation (no. 107), and upper mediastinum (no. 106) were an inherent part of the LAD.^15^

In all patients, reconstruction was done with a gastric tube and a stapled intrathoracic esophagogastrostomy (Ivor Lewis esophagectomy) either as open, hybrid (laparoscopy/thoracotomy), or total-minimally invasive procedure. The surgical approach did not influence the extent of lymphadenectomy. The technical procedure is described in detail elsewhere.^16^^,^^17^

### Pathology Workup

Gross examination of the resected surgical specimen followed a standardized protocol in close cooperation between the operating surgeon and pathologist. After fixation in 4% neutral-buffered formalin overnight, periesophageal and perigastric fat was separately detached from the resected specimen and manually searched for palpable LN (usually > 4 mm in size). Detected LN were counted and put in tissue cassettes for paraffin embedding. Histopathology was performed on serial 5-µm sections stained with hematoxylin and eosin, and the number of retrieved LN and metastases were recorded. Histopathological response to multimodal treatment was assessed using a regression classification system based on the percentage of vital residual tumor cells (VRTCs).^18^ Histopathologic findings were classified according to the UICC TNM classification system.^19^

### Acetone Compression

**Fig. 1:**
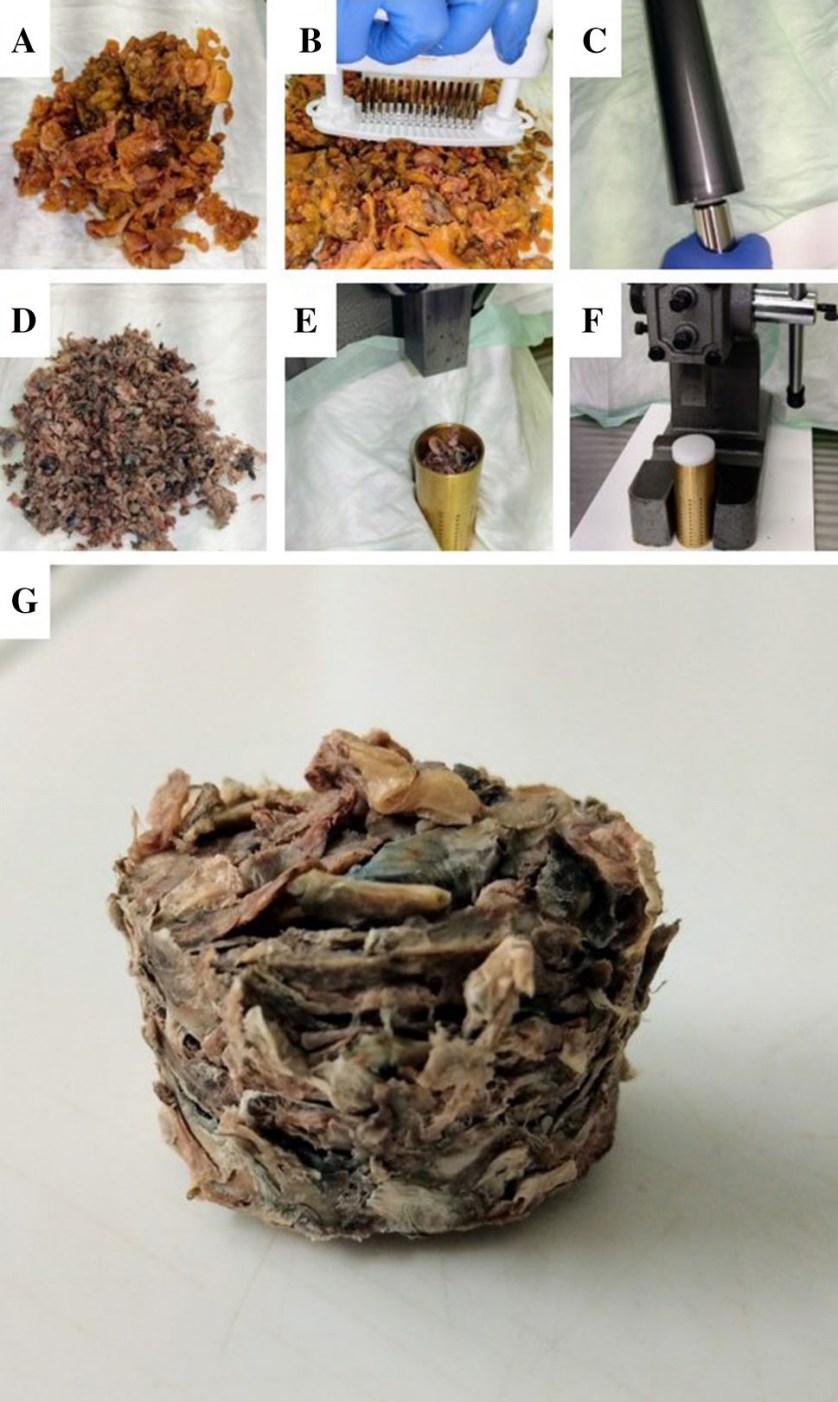
Processing of entire surgically removed fat tissue using acetone compression: **a** The organ-bound or otherwise surgically removed fat is primarily palpated and finely laminated in search of lymph nodes (routine processing). Transfer to acetone. The photo shows acetone-fixed, lamellated fatty tissue. **b**–**d** Further processing of fatty tissue with perforation and rolling of tissue. This results in a first reduction of fatty tissue. **e**, **f** Transfer of fatty tissue into perforated hollow cylinder and compression. **g** Plenty of fat escapes through the perforations, which again contributes to a considerable reduction in volume, allowing entire removed fatty tissue to be transferred into capsules for paraffin embedding to a practicable extent

Complete embedding of remaining fatty tissue was performed by an optimized “acetone compression” protocol (Supplementary Fig. 1). The fatty tissue was isolated, perforated using a meat tenderizer, and incubated in 99% acetone overnight. Compression was conducted by a combination of manual compression using a rolling pin and a mechanical press (RS Pro Arbor press, RS Components, Germany). A custom-build brass tube was used for mechanical compression as previously described (kindly provided by J. Rüschoff, Pathologie Nordhessen, Kassel, Germany). Repetition of the acetone incubation–compression cycle increased efficiency to > 90% weight reduction. The process removes virtually all fat from the tissue. The remaining tissue was manually pulled apart using forceps and embedded in paraffin (Fig. 1). Approximately 1 g compressed tissue fits into a standard tissue cassette, which equals 15–20 g uncompressed tissue.

We placed the fatty connective tissue in acetone, separated by anatomical regions. The surgical specimen was divided into three regions (periosesophageal fat-connective tissue, small-curvature fat-connective tissue, and large-curvature fat-connective tissue). If further lymph node stations were removed, they were sent separately to the Institute of Pathology in a separate container, indicating the anatomical location. This connective tissue was also degreased in a separate acetone storage box and then compressed and processed separately.

### Statistical Analysis

Study patients were stratified according to treatment strategy and divided into three subgroups (group 1: primary surgery; group 2: neoadjuvant CROSS plus surgery; group 3: neoadjuvant FLOT plus surgery). The primary outcome variable was the total number of detected lymph nodes before and after acetone compression. The secondary outcome parameter was the number of metastatic lymph nodes detected with and without acetone compression.

Based on a prospective database, analysis was done retrospectively using GraphPad Prism 8 (GraphPad Software). Statistical analysis was primarily based on descriptive means. Normal distribution was tested using the D’Agostino–Pearson omnibus *K*^2^ test. Statistical differences between lymph node retrieval for different histopathological tissue processing techniques were assessed using the Fisher’s exact test, the Wilcoxon square test, or the independent-samples *t* test as appropriate. *p* values < 0.05 were considered statistically significant.

## Results

### Patient Characteristics

Epidemiologic and histopathologic data of 77 study patients with complete fat embedding using acetone compression are presented in Table 1. Patients were predominantly male (85.7%), and mean age was 62.0 years. Due to locally advanced tumors, the majority of patients (94.8%) received neoadjuvant therapy according to either the CROSS or FLOT protocol.

**Table 1:** Baseline characteristics of 77 study patients

Variable	*N* = 77
Sex (male)	66 (85.7%)
Age (mean, range)	62
BMI (mean, range), kg/m^2^	26.9
Neoadjuvant therapy	
Total	73 (94.8%)
CROSS	44 (57.1%)
FLOT	29 (37.7%)
pT stage	
(y)pT0	7 (9.1%)
(y)pT1	13 (16.9%)
(y)pT2	13 (16.9%)
(y)pT3	39 (50.6%)
(y)pT4	5 (6.5%)
pN stage	
(y)pN0	32 (41.6%)
(y)pN1	27 (35.1%)
(y)pN2	11 (14.3%)
(y)pN3	7 (9.1%)
Complete response, ypT0N0	6/73 (8.2%)

### Histopathologic LN Dissection and Pathological Preparation

We were able to embed the entire removed fat-connective tissue in 9–38 additional tissue capsules by our compression technique (on average 17 additional capsules).

The total and mean number of retrieved LN for the different subgroups using the standard and acetone preparation are summarized in Table 2.

**Table 2:** Detected lymph nodes in 77 patients using standard fat preparation followed by acetone compression with complete embedding of removed fatty tissue

	*N*	LN (manual retrieval)	LN (acetone compression)	*P*-value
Study cohort	77	1781 23.1 (10–43)	3120 40.5 (16–114)	*P* < 0.0001
Primary surgery	4	103 25.8 (17–42)	171 42.8 (28–59)	*P* = 0.124
Neoadjuvant Tx	73	1678 22.9 (10–43)	2949 40.4 (16–114)	
CROSS	44	936 21.3 (10–37)	1667 37.9 (19–60)	*P* < 0.0001
FLOT	29	742 25.6 (11–43)	1282 44.2 (16–114)	*P* < 0.0001
Metastatic LN (in pN positive patients)	45	142 3.2 (0–27)	187 4.2 (1–29)	*P *< 0.0001

*LN* lymph node

**Fig. 2:**
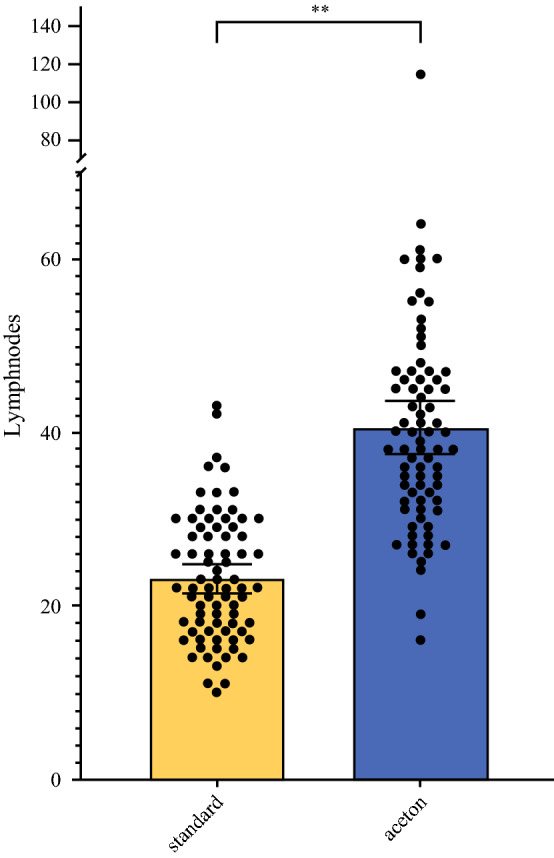
Number of detected lymph nodes before and after using acetone compression in 77 patients following Ivor Lewis esophagectomy

**Fig. 3:**
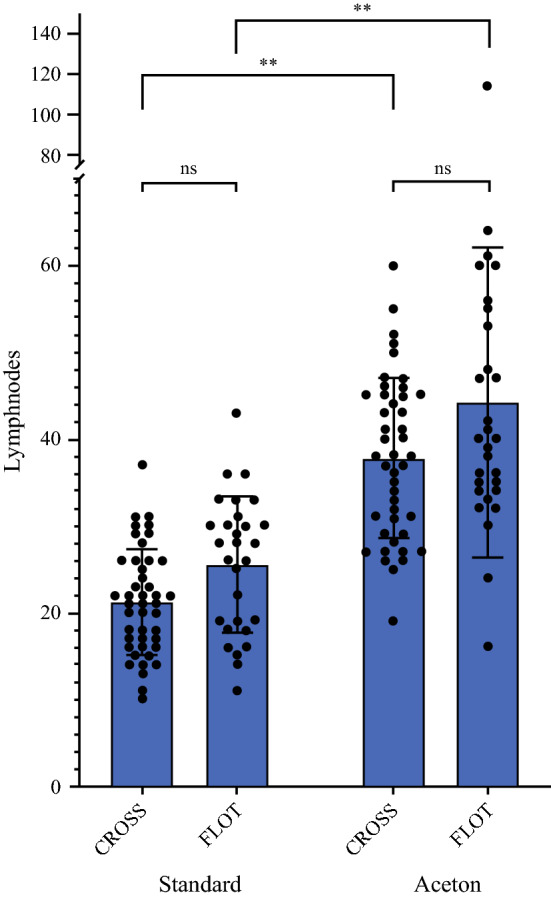
Number of detected lymph nodes before and after using acetone compression in 73 patients receiving neoadjuvant therapy according to the CROSS and FLOT protocol

For the standard pathological workup, a total of 1781 LN with a mean of 23.1 LN (range 10–43 LN; 95% interval 21.5–24.8 LN) could be detected. Additional workup using the acetone technique significantly increased the mean number of detected LN by 17.4 (mean 40.5 LN, range 16–114 LN, 95% 37.5–43.6 LN) (*p* < 0.0001) (Fig. 2). This observation could be demonstrated for patients with primary surgery (*n* = 4) and patients with multimodal treatment (*n* = 73) (Table 2). In the 73 patients who underwent neoadjuvant therapy, the mean number of detected LN before and after acetone compression was 22.9 (range 10–43) and 40.4 (range 16–114), respectively (*p* < 0.0001). This difference was significant for patients following the CROSS protocol (*p* < 0.0001) as well as the FLOT regimen (*p* < 0.0001). Though more LN were histopathologically detected in patients following the FLOT protocol before and after the acetone technique, this difference was not statistically significant when compared with patients following the CROSS protocol (before *p* = 0.324, after *p* = 0.064) (Fig. 3).

Histological features of the tissue were generally well preserved after acetone compression (Supplementary Fig. 2). LN were intact with definable capsules. Blood vessels and small peripheral nerves could be observed and checked for angioinvasion and perineural infiltration. Many LN after acetone compression were < 1 mm in diameter (Supplementary Fig. 2B). LN micrometastases are histomorphologically well recognized and clearly diagnosable. Supplementary Fig. 3 shows a case that was not found to be lymphonodally metastasized after standard preparation (pN0) while complete embedding of fatty tissue revealed a LN micrometastasis (Supplementary Fig. 3 B2).

In the study cohort of 77 patients, the mean number of metastatic LN increased from 3.2 to 4.2 nodal metastases following the acetone compression technique (*p* < 0.0001) (Table 2). Ten patients (13.0%) showed more LN metastases after acetone compression of fatty tissue compared with manual dissection alone. In all ten patients, the additional workup using acetone compression was associated with a change in the primary (y)pN stage. A total of four patients (5.2%) classified as pN0 after standard workup had to be reclassified as pN1 following the acetone technique.

## Discussion

For esophageal cancer, histopathological nodal (pN) status is clearly a variable of outstanding prognostic relevance.^3^^,^^18^ Not only this, but the number of metastatic and total resected LN are recognized to have significant impact on long-term survival.^5^^,^^6^ Therefore, for esophageal adenocarcinoma, national and international guidelines recommend standardized surgical lymphadenectomy of the abdominal and mediastinal compartment with a minimum number of lymph nodes to be resected. This standard allows representative histopathological staging of nodal status.^2^ Despite advances in imaging techniques, preoperative detection of lymph node metastases remains inaccurate and only succeeds in obviously enlarged or bulky lymph nodes. This difficulty in clinical staging can be explained by morphological studies demonstrating the vast majority of resected nodes to be < 4 mm with no significant correlation between size and malignant involvement.^8^ Therefore, the gold standard for nodal staging remains pathological workup of the resected specimen with careful, tactile, and lamellar processing of the fat-connective tissue as an essential histopathological technique for finding surgically removed LN. However, the total number of detectable LN does not only depend on pathologist experience, but is also subject to individual differences, extent of surgical resection, and neoadjuvant treatment. The latter can influence the size and consistency of lymph nodes to such an extent that their palpability is greatly reduced or even impossible. Several publications have addressed the impact of neoadjuvant therapy on surgical lymph node retrieval in rectal and esophageal cancer.^20^^–^22 To increase the accuracy of histopathological nodal staging, several advanced techniques have been proposed, including use of solvents to degrease tissue and injection of dyes into blood vessels to visualize lymph nodes during grossing. Gehoff et al.^23^ presented a technique based on acetone compression of surgically removed fat-connective tissue in neoadjuvant-treated rectal cancer. Another advantage of this method is its flexible applicability. For this study, we were able to preserve lymph node stations of different anatomical localizations, so that after completion of the acetone compression, statements about the final lymph node status of different anatomical stations were possible. For this purpose, removed fat-connective tissue was transferred into different acetone-filled containers, which were compressed and processed separately. This technique has not been investigated in any other tumor entity so far, and the rate of detected LN and its possibly associated pN stage migration is not known.

To the best of the authors’ knowledge, this is the first observational study applying this novel technique in esophageal cancer. The technique is based on degreasing the tissue by a combination of incubation in acetone and mechanical compression, which allows efficient complete embedding. The achieved comprehensive histopathological workup allows retrieval of all contained LN and reduced subjectivity. Here, lymph node numbers before and after acetone compression were compared. In contrast to the standard procedure, this improved tissue processing allows reliable statements on the real number of surgically removed LN.

In this observational study at a high-volume center for esophageal cancer, only patients with adenocarcinoma were included to examine a standardized extent of surgical lymphadenectomy as described above for the Ivor Lewis esophagectomy. As published for comparable patient cohorts, the vast majority of patients presented with locally advanced tumors and received neoadjuvant treatment—either chemoradiation according to the CROSS protocol or chemotherapy alone following the FLOT protocol.^11^,^13^ This treatment strategy is in accordance with national guidelines and is generally accepted as the standard of curative treatment for esophageal adenocarcinoma.^10^

Using standard pathological workup of the specimen, an average of 23.1 LN could be detected in the study, a number comparable with several recent benchmark studies.^24^^,^^25^

After application of the acetone fat-compression technique, a mean of up to 17 additional LN could be diagnosed by light microscopy. This additional LN yield was associated with a pN change in 13% of cases as compared with routine workup. It is important to note that all additional LN metastases were micrometastases with maximum size of 2 mm. Though not an issue of investigation in this study, it can be assumed that the observed change in the UICC stage will also have an effect on oncologic prognosis. This underlines the importance of complete fat tissue workup, since additionally found LN metastases cannot be detected otherwise, neither radiologically nor with standard histological workup.

We examined patients with different body mass index (BMI, cutoff point 25 kg/m^2^) and investigated whether BMI correlates to the number of existing lymph nodes (more fatty tissue with higher BMI accompanied by a higher number of lymph nodes?). This is not the case. There is no correlation between BMI and number of detectable lymph nodes (Supplementary Fig. 4).

In accordance with national and international guidelines, the vast majority of patients included received neoadjuvant treatment. As reported in other studies, the total number of detected LNs following manual preparation was significantly lower following multimodal treatment as compared with patients who underwent primary surgery.^26^ After complete fat embedding using the acetone compression technique, this difference in LN count of 2–3 LN remained unchanged. This is probably due to the fact that neoadjuvant therapy changes the size and consistency of LN, which then become significantly less visible and palpable during standard processing, thus reducing the number of embedded LNs for final analysis. As demonstrated in this study, this inaccuracy can be almost eliminated by the described tissue processing using acetone compression. However, even after complete embedding of fat-connective tissue, the number of detected LN remained lower for patients who underwent multimodal treatment as compared with patients who underwent primary surgery. This is most likely explained by a small proportion of LNs that completely degenerate as a result of neoadjuvant treatment and can no longer be reliably assigned histologically to a LN tissue. After neoadjuvancy according to CROSS (radiotherapy in combination with chemotherapy), an average of 21.3 LN were found after manual search but 37.9 after acetone compression. After neoadjuvancy according to FLOT (combination of cytostatic drugs without radiotherapy), 25.6 and 44.2 LNs were found, respectively. The degeneration or destruction of LN structure is therefore more pronounced after radiotherapy than after chemotherapy alone. In neoadjuvant pretreated rectal carcinoma (also treated with radiotherapy), the phenomenon of low numbers of LNs that are often only poorly detectable has also been known for many years.^20^^–^22

The predominant limitations of this study are its single-center approach and the lack of prognostic data which underline the assumed prognostic significance of the UICC stage migration. A possible disadvantage of the acetone technique is the time required for preparation (about 45 min by a doctor) and additional preparation of tissue blocks by medical technical assistants (about 20 min). Sufficient space must be available to store the various storage boxes (separated according to anatomical region). The odor nuisance caused by acetone is known and regulated in standard operating procedures (SOPs), since acetone is also used in other histopathological contexts.

## Conclusions

The results of this observational study clearly demonstrate that the number of detected LN can be significantly increased by the acetone compression technique, with effects on the UICC stage in more than one in ten patients. As previously shown for rectal cancer, this innovative technique qualifies as a new standard pathologic workup since the number of histopathologically diagnosed LN reflects the real number of resected LN. As a consequence of this examination, tissue compression has already been adopted as a new standard procedure for histopathological processing of removed fat-connective tissue after Ivor Lewis esophagectomy at our high-volume center. As the technique is easy and cost-efficient, it can be applied to standardize and optimize pathological workup in prospective clinical trials. A future multicenter study will investigate effects of optimized histopathological processing after tissue compression with regard to its long-term prognostic relevance.

## Supplementary Information

Below is the link to the electronic supplementary material.

Supplementary material 1 (DOCX 16198 kb)
