# Supplementary material for: Improved Tissue Processing in Esophageal Adenocarcinoma After Ivor Lewis Esophagectomy Allows Histological Analysis of All Surgically Removed Lymph Nodes with Significant Effects on Nodal UICC Stages
Source: Ann Surg Oncol. 2020 Dec 10;28(7):3975–82. doi: 10.1245/s10434-020-09450-1 (PMC8184552; doi:10.1245/s10434-020-09450-1)
Supplement: Supplementary file 1 — Supplementary material 1 (DOCX 16198 kb) [file 10434_2020_9450_MOESM1_ESM.docx]

**Supplementary figure 1:** Overview of optimized acetone compression

After gross examination and manual retrieval of palpable lymph nodes, the remaining fatty tissue is isolated, perforated using a meat tenderizer and incubated in acetone 99% over night (~ 20 h). Compression is achieved by a combination of manual compression using a rolling pin and a mechanical press. The cycle is repeated once with another 8 h incubation step. Overall, the procedure require one additional day for work-up.


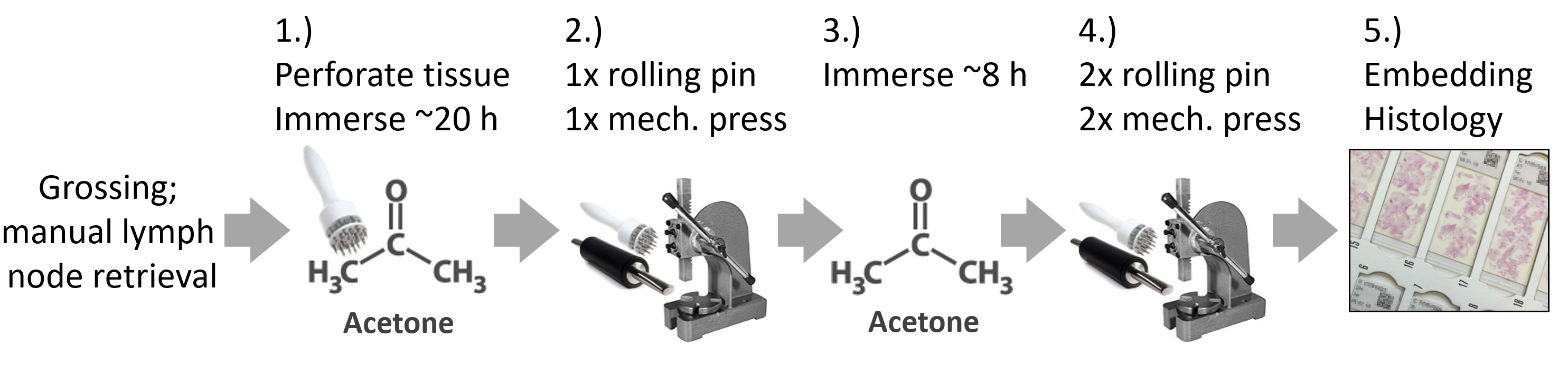


**Supplementary figure 2**: Histology after acetone compression

The histological features of the fatty and connective tissue are generally well preserved after acetone compression. Medium-sized lymph nodes with diameters 1 – 4 mm (A1, A2) as well as small lymph nodes with diameters < 1 mm (B1, B2) are easily located and show intact capsules, marginal sinuses and follicular lymphatic tissue. In additional, blood vessels (C1, C2), small peripheral nerves (D) and inflammatory infiltrates (E) can be examined for tumor infiltrates.


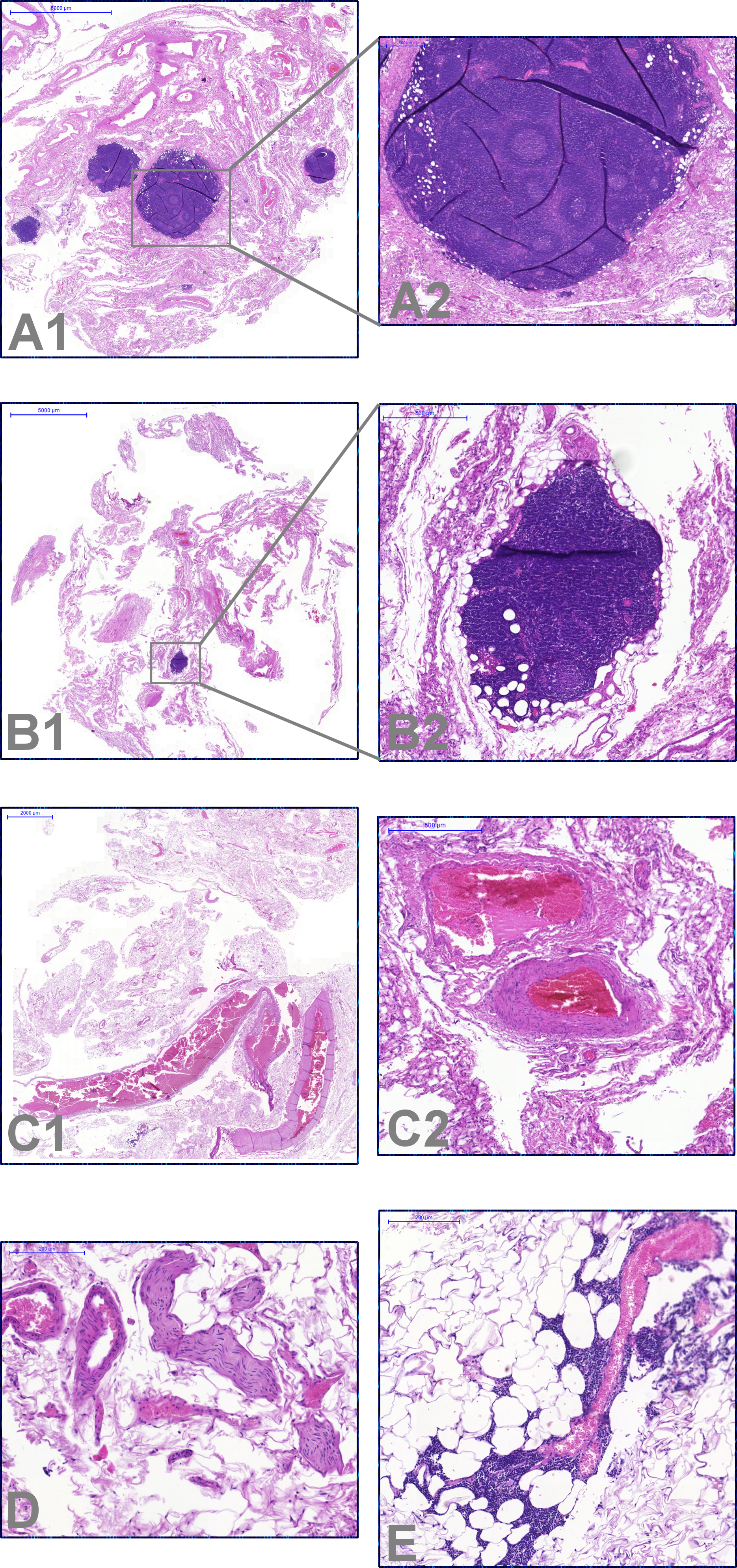


**Supplementary figure 3**: Example of a micrometastasis detected after AC

Examples of lymph nodes of one surgical specimen retrieved by manual dissection (A1, magnified detail: A2) and by acetone compression (B1, magnified detail: B2). In the uncompressed fatty tissue, adipocytes constitute to major volume of the tissue (A2). After compression, the fatty vacuoles have been removed and the remnants of the adipocytes are tightly compressed and appear as a red, eosinophilic mass (B2). The histological architecture of the contained lymph nodes is well preserved and the nodes are easy to spot (long arrow show the lymph node capsule). In this particular case, a single micrometastasis (small arrow) with a diameter of ~ 1 mm was found after AC (B2), causing a TNM-upstage from pN0 to pN1(mi). (Scale bars: A1, B1: 5 mm; A2, B2: 1 mm)


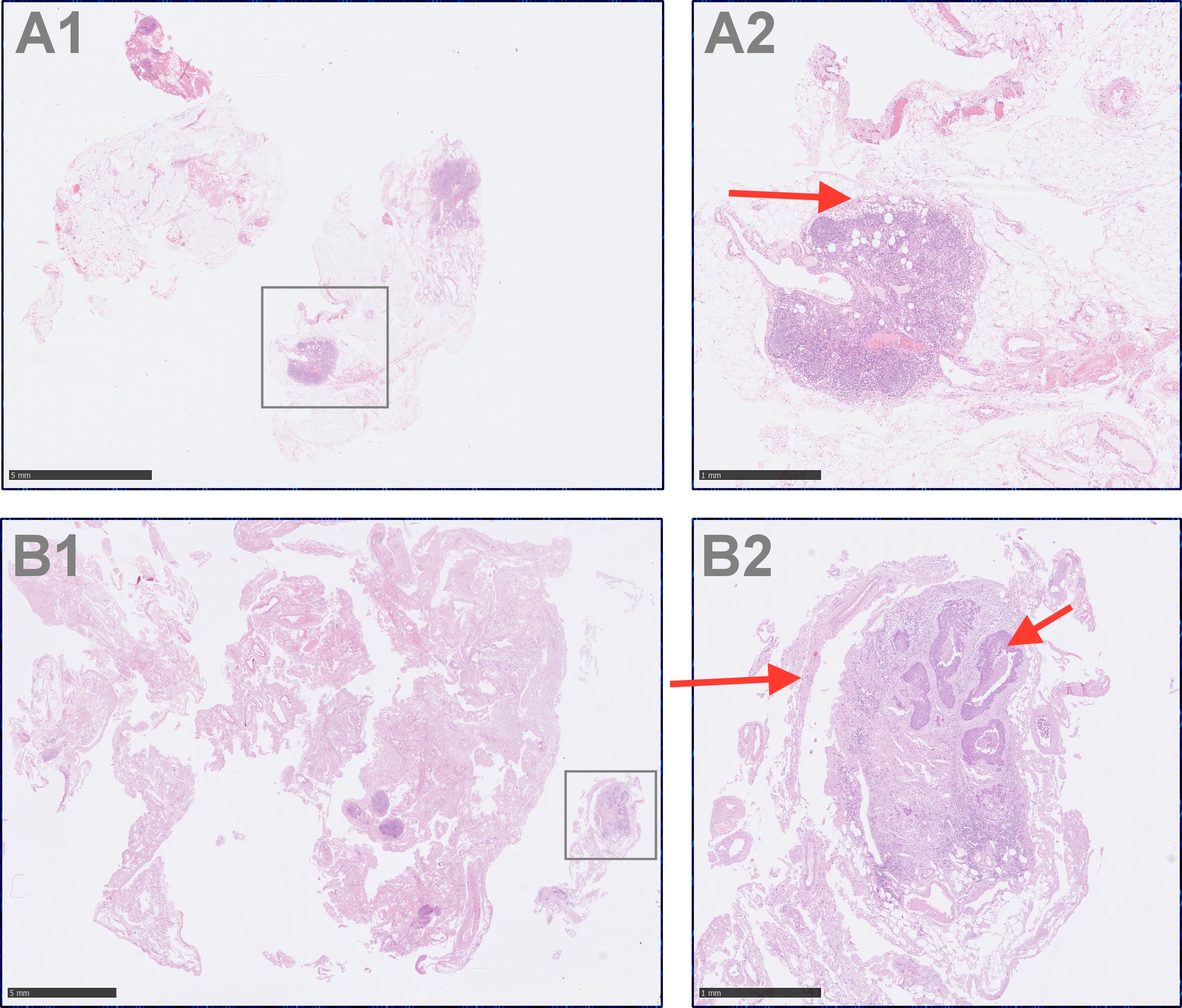


**Supplementary figure 4: BMI and number of LN**

Here the correlation between the body mass index (BMI) and the number of lymph nodes after standard preparation (=LK prim) and after acetone compression is shown. The cut-off value between BMI low versus BMI high was set at BMI 25.
